# Hemoglobins in the genome of the cryptomonad *Guillardia theta*

**DOI:** 10.1186/1745-6150-9-7

**Published:** 2014-05-08

**Authors:** David R Smith, Serge N Vinogradov, David Hoogewijs

**Affiliations:** 1 Department of Biology, Western University, London, Ontario N6A 5B7, Canada; 2 Department of Biochemistry and Molecular Biology, Wayne State University School of Medicine, Detroit, Michigan 48201, USA; 3 Institute of Physiology and Zürich, Center for Integrative Human Physiology, University of Zürich, Winterthurerstrasse 190, Zürich CH-8057, Switzerland

**Keywords:** Protist, Cryptomonads, Hemoglobin, Phylogeny

## Abstract

**Reviewers:**

This article was reviewed by Purificacion Lopez-Garcia and Igor B Rogozin.

Cryptomonads, are a lineage of unicellular and mostly photosynthetic algae, that acquired their plastids through the “secondary” endosymbiosis of a red alga — and still retain the nuclear genome (nucleomorph) of the latter. We find that the genome of the cryptomonad *Guillardia theta* comprises genes coding for 13 globin domains, of which 6 occur within two large chimeric proteins. All the sequences adhere to the vertebrate 3/3 myoglobin fold. Although several globins have no introns, the remainder have atypical intron locations. Bayesian phylogenetic analyses suggest that the *G. theta* Hbs are related to the stramenopile and chlorophyte single domain globins.

## Findings

Endosymbiosis is a fundamental process that has shaped the diversity and evolution of unicellular eukaryotes [1]. “Primary endosymbiosis” - the uptake of a photosynthetic cyanobacterium by an early nonphotosynthetic unicellular eukaryote gave rise to the double membrane-bound plastids of glaucophytes, red and green algae, and land plants [1,2]. Subsequent endosymbioses of primary-plastid-containing eukaryotes by nonphotosynthetic hosts, called “secondary endosymbiosis”, gave rise to much of the present day diversity of protists [3]. Although in most protist lineages, reduction of the endosymbiont nucleus has been completed, a remnant nucleus, the nucleomorph is still present in two protist lineages, the cryptomonads and the chloroarachniophytes [4]. The nucleomorphs of these two groups have independent origins: the cryptomonad plastid and nucleomorph are of red algal ancestry [5,6] whereas the chlorarachniophyte plastids and nucleomorphs are of green algal origin [7,8]. Several cryptophyte genomes have been sequenced, including *Guillardia theta, Bigelowiella natans, Hemiselmis andersenii* and *Cryptomonas paramecium*[7-10]. Here, we report the presence of hemoglobin genes in the host nuclear genome of *G. theta* and on the relationship of the sequences they encode to protist and animal globins*.*

We find 13 globin domains in 9 proteins in the nuclear genome of *Guillardia theta* from the assignments listed on the SUPERFAMILY web site (http://supfam.org), based on a library of hidden Markov models [11,12]. All the sequences were subjected to a FUGUE search [13] (http://www-cryst.bioc.cam.ac.uk), a stringent test of whether a given sequence is a globin [14,15]. Our criteria for accepting a sequence to be a true globin, are the following: a FUGUE Z score >6 (corresponding to 99% probability), the occurrence of a His residue at position F8 and proper alignment of helices BC through G, satisfying the myoglobin fold [16]. Although 7 domains occur in single domain (SD) globins, 6 occur within 2 large (>1000 residues) chimeric proteins, 1060 residues (EKX33177.1) and 1497 residues (EKX39126.1), both of which appear to have a putative cytochrome b5 N-terminal. The 13 globin domains exhibit identity scores ranging from 7 to 70% (Additional file 1). A MAFFT alignment [17] of the 13 domains with sperm whale Mb is shown in Additional file 2. All the *G. theta* Hbs have a His at the proximal position F8, except for the 275 residues globin (EKX43967.1). Interestingly, the latter contains a potential myristoylation site predicted with very high probability, a post-translational modification observed in several vertebrate and invertebrate globins [18,19]. At the distal position E7, the majority of the residues are hydrophobic and at position CD1 all globins contain a Phe. Furthermore, the globin domain D3 of the 1060 residue chimeric protein (EKX33177.1), appears to lack the H helix. Thus, apart from two defective sequences, all the observed *G. theta* globins appear to be fully functional, and their alignment with the sperm whale Mb sequence (Additional file 2), demonstrates clearly their adherence to the canonical Mb-fold [16].

**Figure 1:**
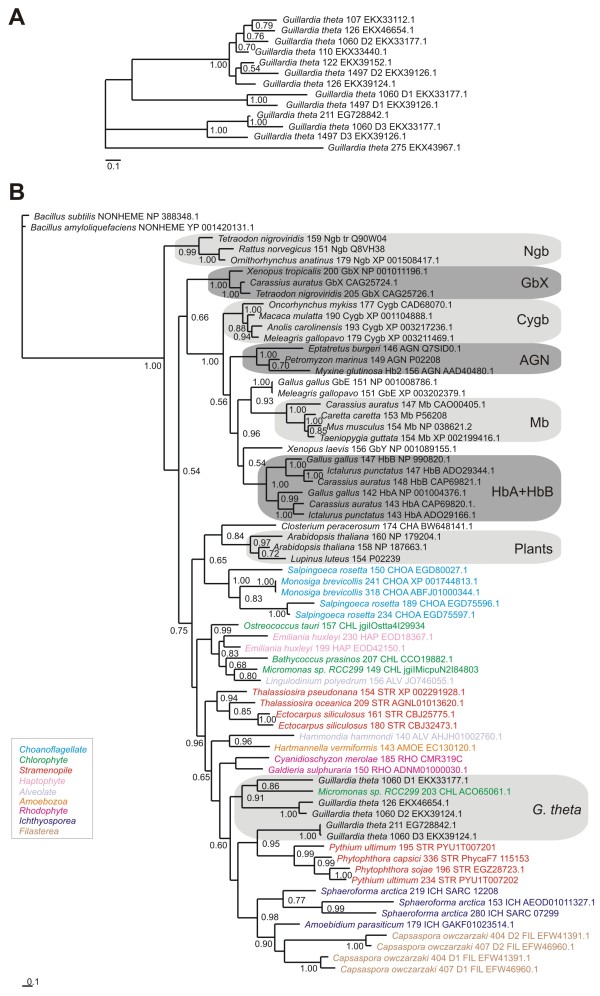
**Phylogeny of *G. theta *Hbs. (A)** Bayesian phylogenetic tree of the 13 globin domains of *G. theta* aligned using MAFFT L-INS-i MSA. Bayesian phylogenetic reconstruction was performed by MrBayes 3.2.2 employing a mixed substitution model. MCMCMC sampling was carried out using 2 independent runs for 1′000′000 generations on the CIPRES web portal [39]. Support values at branches represent Bayesian posterior probabilities (>0.5). **(B)** Bayesian phylogenetic tree based on a Clustal Omega MSA, of *G. theta* Hbs with representative vertebrate, protist and choanoflagellate Hbs, using the two *Bacillus* nonheme globins as outgroup [23]. Bayesian phylogenetic reconstruction was performed by MrBayes 3.2.2 employing a mixed substitution model. MCMCMC sampling was carried out using 2 independent runs for 10′000′000 generations on the CIPRES web portal [39]. All globin sequences are identified by full species name, the number of residues, the abbreviated phylum and family names, and their identification numbers. Support values at branches represent Bayesian posterior probabilities (>0.5). Abbreviations of protist taxons: ALV – Alveolate; AMOE – Amoebozoa; CHOA – Choanoflagellate; CHL – Chlorophyte; FIL – Filasterea; ICH – Ichthyosporea; HAP – Haptophyte; RHO – Rhodophyte; STR – Stramenopile.

A Bayesian phylogenetic analysis [20] of a MAFFT L-INS-i alignment [17] of the 13 *G. theta* globin domains provided an unrooted tree shown in Figure 1A. They form 3 separate clusters: the small SD globins (EKX39152.1, EKX39124.1, EKX33440.1, EKX33112.1 and EKX46654.1) group with the D2 domains of the two chimeric proteins, the D1 and D3 domains form another cluster and the defective globin lacking the F8 His (EKX43967.1) occurs as an outlier. Figure 1B depicts the phylogenetic tree resulting from a molecular Bayesian analysis of a multiple sequence alignment (MSA) of *G. theta* Hbs representative of the three clusters observed in Figure 1A and sequences representing 8 vertebrate globin families (Ngbs, Cygbs, GbX, GbY, GbE, Mb, HbA and HbB), 2 cyclostome Hbs, 5 choanoflagellate Hbs as well as 26 protist sequences, including chlorophytes, haptophytes, stramenopiles, rhodophytes, alveolates, ichthyosporeans and filastereans. We employed Clustal Omega [21] for the MSA and GUIDANCE [22] to assess the quality of the MSA and improve it via removal of low-scoring columns. We used as outgroup either the 2 *Bacillus* nonheme globins [23] or plant 3/3 Hbs, including one LegHb and 2 NsHbs. Although the *Bacillus* nonheme globins have the 3/3 Mb fold, their heme binding cavity is defective due to wider separation of helices. Consequently, we think that they represent the optimal outgroup for globin phylogeny. The sequences used in our analysis are provided in Additional file 3. The animal and protist sequences are widely separated, with the *G. theta* Hbs clustering together surrounded by stramenopile, rhodophyte and chlorophyte sequences. A major concern in globin phylogeny, is the poor statistical support for the nodes occurring in phylogenetic trees, irrespective of the MSA’s employed, the type of phylogenetic analysis and the evolutionary models used. On one hand, the globin sequences are relatively short, and on the other, the low identities found in pairwise alignments of distantly related globins results in the masking of phylogenetic signals coded within the sequences. We sought to extend the aforementioned result by performing additional MSA’s and additional molecular phylogenetic analyses, including Maximum Likelihood (ML) using MEGA version 5.2 [24]. The result of a ML analysis of the same set of sequences as in Figure 1B, aligned using Clustal Omega, shown in Additional file 4, is in broad agreement with the Bayesian tree, despite low bootstrap support. We show Bayesian trees of MSA’s using the same sequences as in Figure 1B and Additional file 3, based on MAFFT L-INS-i (Additional file 5) and MUSCLE [25] (Additional file 6). They are in broad agreement with the Bayesian tree based on the Clustal Omega MSA (Figure 1B). The *G. theta* Hbs again cluster together with several stramenopile Hbs. Furthermore, all Bayesian trees reproduce the known phylogenetic relationships between the vertebrate globins, determined by J. Storz and colleagues [26-28]. A diagram of the latter is provided as Additional file 7.

Although the genes coding for the numerous *G. theta* Hbs are part of the nuclear and not the nucleomorph genomes, their very presence is remarkable in a unicellular organism that has undergone two endosymbiotic events and the succeeding, extensive reductions and processing of the plastid and nucleomorph genomes. The locations of introns in *G. theta* Hbs are also unusual. In vertebrates, intron locations are fairly constant, with two introns inserted at conserved positions B12.2 (intron located between codon positions 2 and 3 of the 12th amino acid of globin helix B) and G7.0 [29]. In contrast, intron locations appear to be highly variable in protostome phyla, e.g. in nematodes [30-32] and in *Chironomus*[33]. In the case of *G. theta* Hbs, we find that introns are absent in 9 out of 13 globin genes. The remaining globins contain intron insertion sites at atypical positions such as B2.0, E1.1, E15.0 and F8.0 for EKX43967.1 (Additional file 2). The D3 globin domains in the two chimeric, multidomain proteins (EKX33177.1 and EKX39126.1) are interrupted by 4 introns: they share the C1.0 and interhelical EF7.0 insertion positions combined with intron insertions at B4.0 and at E6.2 for EKX33177.1, and at G19.0 and H11.0 for EKX39126.1. Absence of introns has also been observed in 3/3 globins from Archaeplastida genomes *C. merolae, O. tauri, M. pusilla, and M. sp. RCC299*[34].

The presence of several globins in the cryptomonad *G. theta* adds yet another puzzle in the pursuit of an explanation for the physiological role of Hbs in microbial eukaryotes. The possible functions of Hbs in bacteria, fungi and protists have been reviewed recently (see references [35-37]). The *G. theta* Hbs are 3/3 globins related to the FHbs and the related single domain globins found in bacteria [37] and protists [35]. Although the functions of metazoan Hbs vary widely, from oxygen transport and storage to enzymatic [38], the latter are obviously more likely in bacteria and in microbial eukaryotes [35]. Our molecular phylogenetic analyses suggest that the *G. theta* Hbs are related to the stramenopile and chlorophyte single domain globins.

## Reviewers’ comments

### Reviewer’s report1: Purificacion Lopez-Garcia (Centre National de la Recherche Scientifique, France)

This discovery note describes the presence of hemoglobin genes in the genome of a cryptomonad species and presents a phylogenetic analysis of those sequences. The observation of hemoglobin genes in cryptomonads may have some interest, although it does not come as a surprise taking into account that globin genes seem universally distributed and have been already detected in very distant eukaryotic lineages, including not only plants and animals but also various divergent protist groups. However, the phylogenetic analyses presented bring little light on the origin and the evolution of cryptophyte globins. Indeed, despite the laudable efforts carried out by the authors to extract some phylogenetic information from these genes, the phylogenetic trees are not well resolved and many nodes are not supported. This means that the remaining phylogenetic signal in these genes is low and, unfortunately, of little use to discuss about globin evolution in eukaryotes.

My major problem with this note is that, despite the very limited phylogenetic information carried out by these genes, the authors try to make sensible conclusions out of it. Unfortunately, apart from being globin homologs, little more can be said from a phylogenetic perspective. Hence, some affirmations seem out of place or meaningless; for instance:

– Abstract and last sentence of manuscript: “phylogenetic analyses suggest that the G. theta Hbs are related to the globin lineage that gave rise to chlorophyte and land plant Hbs and to animal globins, including vertebrate neuroglobins”. Plants and animals belong to two extremely distant eukaryotic lineages, so that this equals saying that G. theta Hbs are eukaryotic. This does not provide any information as to the evolution of cryptomonad Hbs within eukaryotes or as to their proximity to other eukaryotic groups.

Author’s response: *We have altered the last sentences of the Abstract and the manuscript, to reflect the only limited conclusion we can make, namely that the cryptomonad Hbs cluster with chlorophyte and stramenopile Hbs.*

– Page 5 of Findings: “This result illustrates the positive aspect of the available globin phylogenetic trees, namely a consistent and reproducible clustering of certain groups of sequences, despite the typically less than robust statistical node support”. The authors point here to the main problem of their analysis, the poor statistical support, but at the same time, they would like to believe that the clustering is robust, because it seems reproducible. However, the occurrence of low statistical support is incompatible with solid clustering. Values of 0.5 or 0.6 that can be seen in many nodes of the tree shown in Figure 1 would imply that in around 40 to 50% of cases, that node is not recovered by the phylogenetic analysis.

Author’s response: *We agree with the reviewer’s criticism, since we point it out ourselves. We have rewritten the relevant section in Findings. Based on the suggestion by the second reviewer we have performed novel Bayesian analyses with additional outgroup sequences using different MSA’s and according Guidance evaluations. The new tree shown in Figure*1*B based on a Clustal Omega MSA contains reasonable Bayesian posterior probability support values for the clustering of G. theta globins with chlorophyte and stramenopile Hbs as well as convincing support for the lower clade containing all stramenopile, cryptomonad, amoebozoa, filasterea, ichthyosporea and rhodophyte globins used in the analysis. Furthermore, the additional results based on MAFFT and MUSCLE MSA’s with bacterial nonheme globin sequences as outgroup, are in very good agreement with each other, despite the statistical shortcomings. This is as good a result as can be obtained with single MSA trees of highly divergent and short globin proteins from more basal organisms. Although we agree with the reviewer that discussion of phylogenetic relationships between animal neuroglobins, plant and protist Hbs is not appropriate based on our results, we believe that a limited conclusion about the clustering of the G. theta Hbs with chlorophyte and stramenopile Hbs is acceptable.*

The authors must provide a tree with all the complete species names. The phylogenetic tree shown is very difficult to follow and the abbreviations for species that the authors use are not given. They may want to color them as a function of their taxonomic group, instead of (or in addition to) adding phyla letter codes.

Author’s response: *We have altered the presentation of the tree according to the suggestion by the reviewer to improve its readability. Protist taxa were given a color code and species names were written in full in Figure*1*. All species name abbreviations used in the supplemental trees were included in supplemental file 3. In all supplemental trees the color code was adapted accordingly.*

### Reviewer’s report 2: Igor B Rogozin (NIH, United States of America)

The authors found 13 globin domains in the genome of the cryptomonad Guillardia theta. Several globins have atypical intron locations. These are interesting findings.

I am not sure that the tree shown in the Figure 1B is indeed a correctly rooted tree. The authors claimed that they use “Three plant 3/3 Hbs, one LegHb and 2 NsHbs … as outgroup”. I cannot conclude that this is the correct outgroup, this is my impression when I look at the tree (Figure 1B). Thus any conclusions about phylogenetic clustering/nesting may not be supported by this tree. The authors may try some other outgroups in order to confirm that this is the correct rooting and/or provide strong support that they used correct outgroups. Outgrouping is a problem, for example, https://www.blackwellpublishing.com/ridley/tutorials/The_reconstruction_of_phylogeny13.asp.

Author’s response: *We appreciate the reviewer’s concern and agree that rooting might be a problem. Consequently, we have sought sequences other than plant Hbs to use for rooting. In the revised manuscript, we show in Figure*1*B a Clustal Omega MSA rooted with the nonheme globin sequences from Bacillus. Although the sequences have structures that exhibit a 3/3 Mb fold, the heme binding cavity is defective due to the pulling apart of helical strands. We also use the Bacillus sequences to root MAFFT and MUSCLE MSA’s of the same set of sequences as used in Figure*1*B. The resulting Bayesian trees are now Additional files**5**and**6**in the revised manuscript.*

## Abbreviations

Cygb: Cytoglobin; GbE: Globin E, an eye-specific avian globin; GbX: Globin X, found in fish, amphibians and gnathostomes; GbY: Globin Y found in amphibians; Hb: Hemoglobin; HGT: Horizontal gene transfer; LECA: Last Universal Eukaryote common ancestor; LegHb: Leghemoglobin; Mb: Myoglobin; ML: Maximum likelihood; MSA: Multiple sequence alignment; Ngb: Neuroglobin; NsHb: Nonsymbiotic plant Hb; SDgb: Single domain 3/3 globin related to the N-terminal of FHbs.

## Competing interests

The authors declare that they have no competing interests.

## Authors’ contributions

DRS provided the data on intron locations and helped to draft the manuscript. SNV conceived the study, carried out MSA’s and drafted the manuscript. DH conceived the study, performed the phylogenetic analyses and drafted the manuscript. All three authors approved the final version of the manuscript.

## Supplementary Material

Additional file 1 **Similarity matrix based on a MAFFT MSA of the *G. theta* Hbs.** Click here for file

Additional file 2 **A MAFFT alignment of the 13 globin domains of *G. theta *** **with sperm whale Mb.** The Mb fold template consists of predominantly hydrophobic residues at 37 positions, defining helices A through H: A8, A11, A12, A15, B6, B9, B10, B13, B14, C5, CD1, CD4, E4, E7, E8, E11, E12, E15, E18, E19, F1, F4, F8, FG4, G5, G8, G11, G12, G13, G15, G16, H7, H8, H11, H12, H15 and H19. Although the proximal residue at position F8 (P) is always His, the distal residue (D) at position E7 is mostly Met. No introns were observed in Guithe_107_EKX33112.1, Guithe_110_EKX33440.1, Guithe_122_EKX39152.1, Guithe_126_EKX39152.1, Guithe_126_EKX39124.1, Guithe_126_EKX46654.1 and Guithe_211_EG728842.1. The intron locations in the remaining sequences are variable, marked in green for phase 0, yellow for phase 1 and red for phase 2. Click here for file

Additional file 3 **The sequences of the *G. theta* Hbs and other plant, protist and metazoan Hbs used in the phylogenetic analyses.** Click here for file

Additional file 4 **Maximum likelihood tree of the Clustal Omega MSA of *G. theta *** **Hbs with representative vertebrate, protist, choanoflagellate Hbs and plant Hbs using two *Bacillus *** **nonheme globins as outgroup.** ML analysis was performed by MEGA 5.2 under a WAG substitution model. The resulting trees was tested by bootstrapping with 100 replicates. Same sequences as in Figure 1B and Additional file 3. All globin sequences are identified by the first three letters of the genus name and the first three letters of the species name, the number of residues, the abbreviated phylum and family names, and their identification numbers. Support values at branches represent bootstrap percentages (>50) of ML analysis. Abbreviations of protist taxons: ALV – Alveolate; AMOE – Amoebozoa; CHOA – Choanoflagellates; CHL – Chlorophyte; FIL – Filasterea; ICH – Ichthyosporea; HAP – Haptophyte; RHO – Rhodophyte; STR – Stramenopile. Click here for file

Additional file 5 **Bayesian phylogenetic tree based on a MAFFT MSA, of *G. theta *** **Hbs with representative vertebrate, protist, choanoflagellate Hbs and plant Hbs using two *Bacillus *** **nonheme globins as outgroup.** Bayesian phylogenetic reconstruction was performed by MrBayes 3.2.2 employing a mixed substitution model. MCMCMC sampling was carried out using 2 independent runs for 10′000′000 generations on the CIPRES web portal [39]. All globin sequences are identified by the first three letter of the genus name and the first three letters of the species name, the number of residues, the abbreviated phylum and family names, and their identification numbers. Support values at branches represent Bayesian posterior probabilities (>0.5). Abbreviations of protist taxons: ALV – Alveolate; AMOE – Amoebozoa; CHOA – Choanoflagellates; CHL – Chlorophyte; FIL – Filasterea; ICH – Ichthyosporea; HAP – Haptophyte; RHO – Rhodophyte; STR – Stramenopile. Click here for file

Additional file 6 **Bayesian phylogenetic tree based on a MUSCLE MSA, of *G. theta *** **Hbs with representative vertebrate, protist, choanoflagellate Hbs and plant Hbs using two *Bacillus *** **nonheme globins as outgroup.** Bayesian phylogenetic reconstruction was performed by MrBayes 3.2.2 employing a mixed substitution model. MCMCMC sampling was carried out using 2 independent runs for 10′000′000 generations on the CIPRES web portal [39]. All globin sequences are identified by the first three letter of the genus name and the first three letters of the species name, the number of residues and the abbreviated phylum and family names. Support values at branches represent Bayesian posterior probabilities (>0.5). Abbreviations of protist taxons: ALV – Alveolate; AMOE – Amoebozoa; CHOA – Choanoflagellates; CHL – Chlorophyte; FIL – Filasterea; ICH – Ichthyosporea; HAP – Haptophyte; RHO – Rhodophyte; STR – Stramenopile. Click here for file

Additional file 7 **A diagrammatic representation of the phylogeny of vertebrate globins. **Figure one from ref [28]. Click here for file
