# Supplementary material for: Hemoglobins in the genome of the cryptomonad Guillardia theta
Source: Biol Direct. 2014 May 8;9:7. doi: 10.1186/1745-6150-9-7 (PMC4101818; doi:10.1186/1745-6150-9-7)
Supplement: Additional file 1 — Similarity matrix based on a MAFFT MSA of the G. theta Hbs. [file 1745-6150-9-7-S1.docx]

**Guithe_107_EKX33112.1 Vs Guithe_110_EKX33440.1 : 71.03 % id**

**Guithe_107_EKX33112.1 Vs Guithe_122_EKX39152.1 : 71.96 % id**

**Guithe_107_EKX33112.1 Vs Guithe_126_EKX39124.1 : 67.29 % id**

**Guithe_107_EKX33112.1 Vs Guithe_126_EKX46654.1 : 71.96 % id**

**Guithe_107_EKX33112.1 Vs Guithe_211_EG728842.1 : 22.43 % id**

**Guithe_107_EKX33112.1 Vs Guithe_275*_EKX43967.1 : 11.65 % id**

**Guithe_107_EKX33112.1 Vs Guithe_1497_D1_EKX39126.1 : 20.56 % id**

**Guithe_107_EKX33112.1 Vs Guithe_1497_D2_EKX39126.1 : 63.55 % id**

**Guithe_107_EKX33112.1 Vs Guithe_1497_D3_EKX39124.1 : 22.43 % id**

**Guithe_107_EKX33112.1 Vs Guithe_1060_D1_EKX33177.1 : 19.63 % id**

**Guithe_107_EKX33112.1 Vs Guithe_1060_D2_EKX39124.1 : 70.09 % id**

**Guithe_107_EKX33112.1 Vs Guithe_1060_D3_EKX39124.1 : 23.46 % id**

**Guithe_110_EKX33440.1 Vs Guithe_122_EKX39152.1 : 80.91 % id**

**Guithe_110_EKX33440.1 Vs Guithe_126_EKX39124.1 : 79.09 % id**

**Guithe_110_EKX33440.1 Vs Guithe_126_EKX46654.1 : 72.73 % id**

**Guithe_110_EKX33440.1 Vs Guithe_211_EG728842.1 : 26.36 % id**

**Guithe_110_EKX33440.1 Vs Guithe_275*_EKX43967.1 : 9.43 % id**

**Guithe_110_EKX33440.1 Vs Guithe_1497_D1_EKX39126.1 : 20.00 % id**

**Guithe_110_EKX33440.1 Vs Guithe_1497_D2_EKX39126.1 : 68.18 % id**

**Guithe_110_EKX33440.1 Vs Guithe_1497_D3_EKX39124.1 : 28.18 % id**

**Guithe_110_EKX33440.1 Vs Guithe_1060_D1_EKX33177.1 : 20.00 % id**

**Guithe_110_EKX33440.1 Vs Guithe_1060_D2_EKX39124.1 : 77.27 % id**

**Guithe_110_EKX33440.1 Vs Guithe_1060_D3_EKX39124.1 : 27.38 % id**

**Guithe_122_EKX39152.1 Vs Guithe_126_EKX39124.1 : 79.28 % id**

**Guithe_122_EKX39152.1 Vs Guithe_126_EKX46654.1 : 70.27 % id**

**Guithe_122_EKX39152.1 Vs Guithe_211_EG728842.1 : 25.22 % id**

**Guithe_122_EKX39152.1 Vs Guithe_275*_EKX43967.1 : 8.47 % id**

**Guithe_122_EKX39152.1 Vs Guithe_1497_D1_EKX39126.1 : 19.47 % id**

**Guithe_122_EKX39152.1 Vs Guithe_1497_D2_EKX39126.1 : 70.80 % id**

**Guithe_122_EKX39152.1 Vs Guithe_1497_D3_EKX39124.1 : 25.21 % id**

**Guithe_122_EKX39152.1 Vs Guithe_1060_D1_EKX33177.1 : 21.55 % id**

**Guithe_122_EKX39152.1 Vs Guithe_1060_D2_EKX39124.1 : 73.87 % id**

**Guithe_122_EKX39152.1 Vs Guithe_1060_D3_EKX39124.1 : 26.74 % id**

**Guithe_126_EKX39124.1 Vs Guithe_126_EKX46654.1 : 69.05 % id**

**Guithe_126_EKX39124.1 Vs Guithe_211_EG728842.1 : 25.44 % id**

**Guithe_126_EKX39124.1 Vs Guithe_275*_EKX43967.1 : 9.84 % id**

**Guithe_126_EKX39124.1 Vs Guithe_1497_D1_EKX39126.1 : 19.05 % id**

**Guithe_126_EKX39124.1 Vs Guithe_1497_D2_EKX39126.1 : 66.67 % id**

**Guithe_126_EKX39124.1 Vs Guithe_1497_D3_EKX39124.1 : 24.60 % id**

**Guithe_126_EKX39124.1 Vs Guithe_1060_D1_EKX33177.1 : 22.95 % id**

**Guithe_126_EKX39124.1 Vs Guithe_1060_D2_EKX39124.1 : 72.22 % id**

**Guithe_126_EKX39124.1 Vs Guithe_1060_D3_EKX39124.1 : 23.00 % id**

**Guithe_126_EKX46654.1 Vs Guithe_211_EG728842.1 : 22.81 % id**

**Guithe_126_EKX46654.1 Vs Guithe_275*_EKX43967.1 : 8.20 % id**

**Guithe_126_EKX46654.1 Vs Guithe_1497_D1_EKX39126.1 : 19.84 % id**

**Guithe_126_EKX46654.1 Vs Guithe_1497_D2_EKX39126.1 : 60.32 % id**

**Guithe_126_EKX46654.1 Vs Guithe_1497_D3_EKX39124.1 : 22.22 % id**

**Guithe_126_EKX46654.1 Vs Guithe_1060_D1_EKX33177.1 : 22.13 % id**

**Guithe_126_EKX46654.1 Vs Guithe_1060_D2_EKX39124.1 : 69.84 % id**

**Guithe_126_EKX46654.1 Vs Guithe_1060_D3_EKX39124.1 : 23.00 % id**

**Guithe_211_EG728842.1 Vs Guithe_275*_EKX43967.1 : 9.65 % id**

**Guithe_211_EG728842.1 Vs Guithe_1497_D1_EKX39126.1 : 18.10 % id**

**Guithe_211_EG728842.1 Vs Guithe_1497_D2_EKX39126.1 : 21.55 % id**

**Guithe_211_EG728842.1 Vs Guithe_1497_D3_EKX39124.1 : 60.17 % id**

**Guithe_211_EG728842.1 Vs Guithe_1060_D1_EKX33177.1 : 14.41 % id**

**Guithe_211_EG728842.1 Vs Guithe_1060_D2_EKX39124.1 : 23.68 % id**

**Guithe_211_EG728842.1 Vs Guithe_1060_D3_EKX39124.1 : 97.75 % id**

**Guithe_275*_EKX43967.1 Vs Guithe_1497_D1_EKX39126.1 : 10.94 % id**

**Guithe_275*_EKX43967.1 Vs Guithe_1497_D2_EKX39126.1 : 14.29 % id**

**Guithe_275*_EKX43967.1 Vs Guithe_1497_D3_EKX39124.1 : 10.37 % id**

**Guithe_275*_EKX43967.1 Vs Guithe_1060_D1_EKX33177.1 : 12.20 % id**

**Guithe_275*_EKX43967.1 Vs Guithe_1060_D2_EKX39124.1 : 7.87 % id**

**Guithe_275*_EKX43967.1 Vs Guithe_1060_D3_EKX39124.1 : 9.43 % id**

**Guithe_1497_D1_EKX39126.1 Vs Guithe_1497_D2_EKX39126.1 : 14.62 % id**

**Guithe_1497_D1_EKX39126.1 Vs Guithe_1497_D3_EKX39124.1 : 15.91 % id**

**Guithe_1497_D1_EKX39126.1 Vs Guithe_1060_D1_EKX33177.1 : 54.84 % id**

**Guithe_1497_D1_EKX39126.1 Vs Guithe_1060_D2_EKX39124.1 : 16.92 % id**

**Guithe_1497_D1_EKX39126.1 Vs Guithe_1060_D3_EKX39124.1 : 18.27 % id**

**Guithe_1497_D2_EKX39126.1 Vs Guithe_1497_D3_EKX39124.1 : 25.38 % id**

**Guithe_1497_D2_EKX39126.1 Vs Guithe_1060_D1_EKX33177.1 : 19.35 % id**

**Guithe_1497_D2_EKX39126.1 Vs Guithe_1060_D2_EKX39124.1 : 61.72 % id**

**Guithe_1497_D2_EKX39126.1 Vs Guithe_1060_D3_EKX39124.1 : 23.53 % id**

**Guithe_1497_D3_EKX39124.1 Vs Guithe_1060_D1_EKX33177.1 : 19.69 % id**

**Guithe_1497_D3_EKX39124.1 Vs Guithe_1060_D2_EKX39124.1 : 21.37 % id**

**Guithe_1497_D3_EKX39124.1 Vs Guithe_1060_D3_EKX39124.1 : 56.60 % id**

**Guithe_1060_D1_EKX33177.1 Vs Guithe_1060_D2_EKX39124.1 : 21.31 % id**

**Guithe_1060_D1_EKX33177.1 Vs Guithe_1060_D3_EKX39124.1 : 14.43 % id**

**Guithe_1060_D2_EKX39124.1 Vs Guithe_1060_D3_EKX39124.1 : 21.90 % id**
