# Supplementary material for: Hemoglobins in the genome of the cryptomonad Guillardia theta
Source: Biol Direct. 2014 May 8;9:7. doi: 10.1186/1745-6150-9-7 (PMC4101818; doi:10.1186/1745-6150-9-7)
Supplement: Additional file 2 — A MAFFT alignment of the 13 globin domains of G. theta with sperm whale Mb. The Mb fold template consists of predominantly hydrophobic residues at 37 positions, defining helices A through H: A8, A11, A12, A15, B6, B9, B10, B13, B14, C5, CD1, CD4, E4, E7, E8, E11, E12, E15, E18, E19, F1, F4, F8, FG4, G5, G8, G11, G12, G13, G15, G16, H7, H8, H11, H12, H15 and H19. Although the proximal residue at position F8 (P) is always His, the distal residue (D) at position E7 is mostly Met. No introns were observed in Guithe_107_EKX33112.1, Guithe_110_EKX33440.1, Guithe_122_EKX39152.1, Guithe_126_EKX39152.1, Guithe_126_EKX39124.1, Guithe_126_EKX46654.1 and Guithe_211_EG728842.1. The intron locations in the remaining sequences are variable, marked in green for phase 0, yellow for phase 1 and red for phase 2. [file 1745-6150-9-7-S2.docx]

**nn........................................C.....**

**aaaaaaaaaAaaAAaaAa.bbbbbBbbBBbbBBbbcCcccccDddD..**

**Mb fold .......|..||..|.......|....||..||...|.....|..|**

**1a6m ---------------------vLsegeWqlVlhVWakV--eadvagHGqdILIrLFkshpeTlekFdrF**

**Guithe_107_EKX33112.1 -------------------------------EMIYDCLFKLAPNATHLF------T-------**

**Guithe_110_EKX33440.1 ----------------------------ELGEMVYDHLFRLAPNVASLF------T-------**

**Guithe_122_EKX39152.1 ---------------------------AHLGEMVYDHLFKLAPNVTSLF------T-------**

**Guithe_126_EKX39124.1 ------------IVRDCWDIIMEHYEPSDLGEMVYDHLFRLAPNVASLF------T-------**

**Guithe_126_EKX46654.1 ------------LVRDSWDTISEKYTASDLGGMIYDGLFKLAPSAASLF------N-------**

**Guithe_211_EG728842.1 ------------------------TDIEDLGSIFWKHLNDESPEQTHLF------R-------**

**Guithe_275*_EKX43967.1 SSIIGEDDKLVEDSWKDC--ENNLQEYIDKFVLRIVASNSRSRGIF------TVDEESRV**

**Guithe_1060_D1_EKX33177.1 ------------SWRKLLRKVSYADLGLSIYESV-RDVDELEPLF------RF------**

**Guithe_1060_D2_EKX33177.1 ---GSTLSVVRDCWESILEQYTPADLGELIYDQLFKLAPNVASLF------T-------**

**Guithe_1060_D3_EKX33177.1 ---TSRGELILEHWQEVRVNTDIEDLGSIFWKHLNDESPEQTHLF------R-------**

**Guithe_1497_D1_EKX39126.1 ----LHSARIASSWTELVKKSDYAEIGRRIYGSV-KANDTLEPLF------RF------**

**Guithe_1497_D2_EKX39126.1 ------LGLVRECWDSICEQYTTNELGEMVYDHLFKMAPNLTMLF------T-------**

**Guithe_1497_D3_EKX39126.1 ---NGYNDLVLSSWDIVRQRTEVQELGEKFWKYLNCMSPEQTNLF------R-------**

**.............D...............................P.....F.**

**dddddddeeeEeeEEeeEEeeEeeEEe...........FffFfffFff...G.**

**Mb fold ...|..||..||..|..||............|..|...||....|.**

**1a6m SedLkkHGvtVLtaLGaILkkkghHea---eLkplAqsHAtkhkI**

**Guithe_107_EKX33112.1 -KPREYMAIKMGDTLGMLVSFADDPDDMKKQVASLGLRH-VKYNV**

**Guithe_110_EKX33440.1 -KPREYMAVKMGDTLGMLVSFADDPENMKQQVSWLGIRH-VQYNV**

**Guithe_122_EKX39152.1 -KSREYMAIKMGDMLCMLVSFADDPDNMKQQVSWLGLRH-VNYKV**

**Guithe_126_EKX39124.1 -QPRVYMAVKMGDMLGMLVSFADDPDNMKQQVSCLGLRH-VKYRV**

**Guithe_126_EKX46654.1 -KPRDYMAVKMGDTLGMLVSFADEPDDMKQQVAWLGLRH-VNYHV**

**Guithe_211_EG728842.1 -RSFTMWGKLLQHIMEMLLLSLAEPETFFEQLFELTIRH-IRYGV**

**Guithe_275*_EKX43967.1 EESSKAKATELFAAVGRSIAGLQDCEKCVEI-----LKE-YKFGV**

**Guithe_1060_D1_EKX33177.1 -TNRVVQGTKFVDMLSSIVDNIHSPAEIYVKIADLAPLH-HRKGV**

**Guithe_1060_D2_EKX33177.1 -KPREVMAIKMGNTLGTLVSFADDPESMKQQVTWLGVRH-VLYNV**

**Guithe_1060_D3_EKX33177.1 -RSFTMWGKLLQHIMEMLLLSLAEPETFFEQLFELTIRH-IRYGV**

**Guithe_1497_D1_EKX39126.1 -TNQTVQGTKFVDMLSSIVENINNPQTIFEKVNELAPMH-HRKGV**

**Guithe_1497_D2_EKX39126.1 -KPRSYMAVKMGDMLSMLVSFADSSESMKQQISWLGLRH-VKYKI**

**Guithe_1497_D3_EKX39126.1 -RSLSMWGHLLHHIVNMLLISITDPEEYYDLMFELTIRH-IRYGV**

**ggggGggGggGGGgGGggg.....hhhhhhHHhhHHhhHhhhHhhhhhhh**

**Mb fold ....|..|..|||.||..............||..||..|...|.......**

**1a6m PikyLefISeAIihvLhsrhpgdFgadAqgAMnkALelFrkdIaakYkelgyqg--**

**Guithe_107_EKX33112.1 RPHHIPLIAPVIVNVLAEACGEAWSEEIEQAWSTVIHMVCQNMVE-----------**

**Guithe_110_EKX33440.1 RPHHIPLIGPVFMNVLADVSGEEWTEDVEKAWGIVFKMVCDNMSE-----------**

**Guithe_122_EKX39152.1 RPHHIPLMGPVFMTVLAEASGEYWTEEMEKCWGIVFNMVCENMSEAIQDGEDYALS**

**Guithe_126_EKX39124.1 RPHHIPLMGPVFMSVLSEAAGPDWTPEVEKAWGVVFSMVCESMSE-----------**

**Guithe_126_EKX46654.1 RPHHIPLIGPVIMNALADAAEDAWTEEVEKSWGTIFRMVCENMAE-----------**

**Guithe_211_EG728842.1 RPEYLAPFGTALLLTLEEILKDKWDDRAEAVWKEVWKRAANSMSRGLSL-------**

**Guithe_275*_EKX43867.1 KPEQFLEIADIVSAVVCENGNDS----LKSAWRISVYKTVQKLEDRLRQEYEGLSR**

**Guithe_1060_D1_EKX33177.1 RGSQMPLMQEIVMRVFDSTLGDDMLEEEKKAWLWMWAFLTKALDQSLKEV------**

**Guithe_1060_D2_EKX33177.1 RPHHIPLIGPVIMNALSEAAGAMWTPEVEKSWGIVIKMVCENMAE-----------**

**Guithe_1060_D3_EKX33177.1 PEYLAPFGTALLLTLEEVE-------------------------------------**

**Guithe_1497_D1_EKX39126.1 KAAHMPIMKGIIVSLLKHVLGDEFTNEDEEAWNWIWQYLTQILDQSL---------**

**Guithe_1497_D2_EKX39126.1 RPHHIPLMGPVFLAVVAEAAGVHWSQDTEKAWSVLFNMVCVNMADAI---------**

**Guithe_1497_D3_EKX39126.1 RSEYLNPFGNALFATFEEILSDVWEEKTTKAWKLVWKRATCNMSRGLNMGGNA---**
