# Supplementary material for: Hemoglobins in the genome of the cryptomonad Guillardia theta
Source: Biol Direct. 2014 May 8;9:7. doi: 10.1186/1745-6150-9-7 (PMC4101818; doi:10.1186/1745-6150-9-7)
Supplement: Additional file 3 — The sequences of the G. theta Hbs and other plant, protist and metazoan Hbs used in the phylogenetic analyses. [file 1745-6150-9-7-S3.docx]

>Bacsub_NONHEME_NP_388348.1 (Bacillus subtilis)

SYQLTDQVYENISKEYIDILLLSVKDENAAESQISEAEFWKRLYTKMNDKRLPDQESTIDFSPIFNQYSISWEKTVSLQKIALQELPLIPVFENITVMPLVGTIDMENLLNGVVKHRQVVLDITGVPVVDTMVAHHIIQASEAVRLVGAKIRPEIAQTIVNLGIDLSQVITKNTLQKGIQTAL

>Bacamy_NONHEME_YP_001420131.1 (Bacillus amyloliquefaciens)

DYQLTNHICENICKDYIDILLLSTKNDEATEEQISESEFWKLLYETMVDLNMADQDRAIDFNPILNQYSISWEKTVTLQKIALQELPLIPVFEHVTVMPLVGTIDMENLLNGVVKHRQVVLDITGVPVVDTMVAHHIIQASEAVRLVGAKIRPEIAQTIVNLGIDLSQVITKNTLQKGIQTAL

>Guithe_126_EKX46654.1 (Guillardia theta)

LVRDSWDTISEKYTASDLGGMIYDGLFKLAPSAASLFNKPRDYMAVKMGDTLGMLVSFADEPDDMKQQVAWLGLRHVNYHVRPHHIPLIGPVIMNALADAAEDAWTEEVEKSWGTIFRMVCENMAE

>Guithe_211_EG728842.1 (Guillardia theta)

TDIEDLGSIFWKHLNDESPEQTHLFRRSFTMWGKLLQHIMEMLLLSLAEPETFFEQLFEL

TIRHIRYGVRPEYLAPFGTALLLTLEEILKDKWDDRAEAVWKEVWKRAANSMSRGLSL

>Guithe_1060_D1_EKX33177.1 (Guillardia theta)

SWRKLLRKVSYADLGLSIYESVRDVDELEPLFRFTNRVVQGTKFVDMLSSIVDNIHS

PAEIYVKIADLAPLHHRKGVRGSQMPLMQEIVMRVFDSTLGDDMLEEEKKAWLWMWAFLTKALDQSLKEV

>Guithe_1060_D2_EKX39124.1 (Guillardia theta)

GSTLSVVRDCWESILEQYTPADLGELIYDQLFKLAPNVASLFTKPREVMAIKMGNTLGTLVSFADDPESMKQQVTWLGVRHVLYNVRPHHIPLIGPVFMNVLSEAAGAMWTPEVEKSWGIVIKMVCENMAE

>Guithe_1060_D3_EKX39124.1 (Guillardia theta)

TSRGELILEHWQEVRVNTDIEDLGSIFWKHLNDESPEQTHLFRRSFTMWGKLLQ

HIMEMLLLSLAEPETFFEQLFELTIRHIRYGVRPEYLAPFGTALLLTLEEVE

>Aratha_160_NP_179204.1 (Arabidopsis thaliana)

MESEGKIVFT EEQEALVVKS WSVMKKNSAE LGLKLFIKIF EIAPTTKKMF SFLRDSPIPA

EQNPKLKPHA MSVFVMCCES AVQLRKTGKV TVRETTLKRL GASHSKYGVV DEHFEVAKYA

LLETIKEAVP EMWSPEMKVA WGQAYDHLVA AIKAEMNLSN

>Aratha_158_NP_187663.1 (Arabidopsis thaliana)

MGEIGFTEKQ EALVKESWEI LKQDIPKYSL HFFSQILEIA PAAKGLFSFL RDSDEVPHNN

PKLKAHAVKV FKMTCETAIQ LREEGKVVVA DTTLQYLGSI HLKSGVIDPH FEVVKEALLR

TLKEGLGEKY NEEVEGAWSQ AYDHLALAIK TEMKQEES

>Luplut_154_P02239 (Lupinus luteus)

MGVLTDVQVA LVKSSFEEFN ANIPKNTHRF FTLVLEIAPG AKDLFSFLKG SSEVPQNNPD

LQAHAGKVFK LTYEAAIQLQ VNGAVASDAT LKSLGSVHVS KGVVDAHFPV VKEAILKTIK

EVVGDKWSEE LNTAWTIAYD ELAIIIKKEM KDAA

>Cloper_174_CHA_BW648141.1 (Closterium peracerosum)

FPRSIIIMGSLGSPAEFVLSSEQVQLIKDSFKAKLQADV

PYHAMTLFINLFEIVPEAKALISFTRDYTGPMRENEGLQAHATTVLKKVVNIATNLDNEK

QVEMFSKSLAELGGKHIGYGVKLKHAAKLRDAFVLTMAQGMGDTWSGDVQAAWIAAYDVI

EQMFVVGLVGLAAPK

>Amopar_179_ICH_GAKF01023514.1 (Amoebidium parasiticum)

SLDRGSLDSADAVLVQMLQSEHKRVVQHTWQLAISEKKSKSVTPVAELVESFYNKLFIMM

PETKLMFNNNTFHQTRAMSGALSRMMQYLDNGEQLRDQMQELAATHVHIGVTAEMFDAFG

KALIAAIAERIGADWTPAMQTAWENAYT

>Spharc_153_ICH_AEOD01011327.1 (Sphaeroforma arctica)

LHVYSFDFSRASWRCVCTPRDSQDCFPLLEFSDKFYQALFTLRPETKILFGSMSLQGLKLSKMLHMI

VDQVEFLVNFDVGEIEGEKEKSQFKRMVQTHKRVGITREMLICGGQVRFSVYLNILYSIYAFGNRSS

IVLSSAWCEVLVRISSAGD

>Spharc_219_ICH_SARC_12208 (Sphaeroforma arctica)

MSLTPRQCEMIKSSWKEASQGGKPTEFRALRFVMDFYSHLFDLAPSTKSMFKGGMANQGK

ALVGMLDIVVNHIDSLATIKGDVELLGQRHAKYGVTSNMYVTAGRALVMALAPRIPDDED

KPECASAWMDAYSFLASIMCNAAGDKL

>Spharc_280_ICH_SARC_07299 (Sphaeroforma arctica)

MFILRYSFYSITNSESWKNALKSQNKADKFPSRAFADSFYHYLFITDPSVKVLFAGGLFV

QSKKLANMLNMLVNEIPKIERGHMSPEFERNMESLVTVHHTLAIGRDRFISGGRSLIHAI

VNRLATCECTFAYLDSRNETHLEKLCSSE

>Monbre_241_CHOA_XP_001744813.1 (Monosiga brevicollis)

PSFDPVAVKLARKQWKRVVQLVPNWHEVFFSYLFERAPYA

RTLFPFDVDRLQGNSSLAEHAKRVGQALETALQGLFEYYSLVEVLEKLGRRHFKYGVEPE

HIDLFEETFYKTLAIGLGKKWNPEARRAWEIVCGLILSPIRTGILQARTKANHL

>Monbre_318_CHOA_ABFJ01000344.1 (Monosiga brevicollis)

VAVKLARKQWKRVVQLVPNWHEVFFSYLFERAPY

ARTLFPFDVDRLQGNSSLAEHAKRVGQALETALQGLFEYYSLVEVLEKLGRRHFKYGVEP

EHIDLFEETFYKTLAIGLGKKWNPEARRAWEIVCGLILSPIRTGILQ

>Salros_150_CHOA_EGD80027.1 (Salpingoeca rosetta)

MDDSAMKITQESWAMVEREIPNWTDIFYDKMFSDPNIAKLFPFSAGDFKTNEKFQTHTQKVRDTMHTAMTSIREFEKLGPVLKKMGERHADYGVIPEHSVNFKEAFLHTLKTGYGDKWNEDLDDAWNQCVDALLEPFEDGLNEALAAKNQ

>Salros_189_CHOA_EGD75596.1 (Salpingoeca rosetta)

MHATATTPSARPARALIHAASADKASDSTAKMPGQQVLVREQGGQGRPHFVPHDEMRLDMEQLKIALGSWTAVVELVPTWHEVFFAELFQAHPETERLLYSSDKSKSWNERHMARVGKSVGDVIKSLSNYDDVIEHLTTGEPHEQACCLTDGYVIGTGLGNTPRSLWLACGSTGFVHQRFSLDLGCWTW

>Salros_234_CHOA_EGD75597.1 ((Salpingoeca rosetta))

VPHDEMRLDMEQLKIALGSW

TAVVELVPTWHEVFFAELFQAHPETERLLYSSDKSKSWNERHMARVGKSVGDVIKSLSNYDDVIEHLTALGTRHARYGLHVDQLDLFINAFLWTLGAGLGDSWDHSVKKAWMHVLPFILSPLKSGLVVARTLRNDYNTSG

>Capowc_404_D1_FIL_EFW41391.1 (Capsaspora owczarzaki)

LLRHETRDVIKSTWALAIQKQDE

ADVTPVATFVNVFFGKLFELCPETRLVFGQDLSLQGKSLSSVLTGMLEFVVHPKKLTTQV

KSLAVKHVGLGITPDMFDAFGAALVYTIKTRIGKVWSPQTERVWVDAYGGVNNIITQQMS

RVTLNSQD IPPWMI

>Capowc_404_D2_FIL_EFW41391.1 (Capsaspora owczarzaki)

QRYQEHHAVLHASWVKATEGDNGDAVLTGFLVRLQSNNPQATLIYE

RADPRMRKVIIWTAVSKILDCMQNPRSLRKELKPLGQSHAKMGVTGPMLDSFGVALRAVL

KDVLKARYTTETDMVWRRCYRLFSVQFLASIEAESKGKGVG

>Capowc_407_D1_FIL_EFW46960.1 (Capsaspora owczarzaki)

LLRHETRDAIQSSWALAIQKHDDHD

VTPVATFVNILFAKLFEVCPETRLVFGHDMVRQGKSLSSILTGMLEFVVHPKKLQSQVKR

LAHMHVGLGVTPDMFEAFGFSLLYTIRVRIGSAWNQQIERVWVDTYGGVSNILSQHMSHI

TLD ANDIPSWM

>Capowc_407_D2_FIL_EFW46960.1 (Capsaspora owczarzaki)

VSRYLEHHAVMMATWKEATDADSGERVLNGFFTRLQVNDPQASHIYETA

DARMRKVIIWSAVTKILDCLENPRSLRKELKPLAQSHAHMGVTPQMIDAFGTSLCAVLKE

VLQEKYTTEVDVVWRRCFRLFAAQFSLSLERYSKSKN

>Batpra_207_CHL_CCO19882.1 (Bathycoccus prasinos)

MGFFSKFAKKEQQPSSSDKAPAGTTSGDDDEKKERKAEKLASKQPGKQAAASREEIDYVQ

STWQQIFPMSGPNKLSSEDLDAKKTAVGVLLFREIFSLAPGALALFSFKDVEDVYESPML

KAHGKAVVGAVDAAVHLLDDVSKLVPILEELGQFHNKKNIVGAHYD

>MicrRCC299_149_CHL_jgi|MicpuN2|84803 (Bathycoccus prasinos)

MSVEPNHVQEVENSWNKVAALGVENVGVLLFKNIFTIAPEALELFSFRNEPNLYDSLTLK

AHGVNVVNTVGKAVAGLREFYTLVPALAALGERHVEYGILEPHYDVVGKALLMTLEQGLG

DAFTPQVKEAWTIVYEAVAVTMKGDNYKK

>MicrRCC299_203_CHL_ACO65061.1 (Bathycoccus prasinos)

MVALAPFLAIFGIKRVSKFRR RWTERKRLVIQSSWAALLSAHGNDRMATGSKIFRKLFTG

DTAVLRLFPFRHQARTLFVSAPFKLHAKLFVDTMTELIANLHDLEKVERDVRELGKRHLT

YGVQPAHFDAMGEALIAVLDESCHHPSDEVTLDKEERDAWLGFWGFIAKETQRGRKGIIA

SNPGS

>Osttau_­157_CHL_jgi|Ostta4|29934| (Ostreococcus tauri)

MVDVDAVQRTWALVATSDDAIDAAGKAFFSKIFEIAPGAIELFASFKDVPEEKRYESPGF

VRHARSVMTTVGVAVQGLRDLDALVPVLEKLGARHLGYGVQEAHYDVVGEALLKTLESAL

GDEWTPAVREAWTQTYGVVKTAMIRGATEFERKNLDA

>Cyamer_185_RHO_CMR319C (Cyanidioschyzon merolae)

EPQQ ESIAIVQSTWSQAVQQRERL GQVFYDRLFA LYPELQPMFR SDPALQRIRL

VDMVDAGVKL LNSRRDLEQALRDLGKRHVK YGTQEEQYPI VGENLLHALE

SILGSKHFSE DMRKAWLDVY AYWSSVMLEGAREAQ

>Galsul_150_RHO_ADNM01000030.1 (Galdieria sulphuraria)

SGPEKYVVQLVMVRQTLNFEEIQIVQQSWNK

LEDRQYLIGEAFYHSLFETYPSVKPLFRSDMEKQKRLLIHMINKGVKLLNDIDKLESALS

SLGKRHIKYGVKEEHFPCVGETLLKVLKQFLGDEFDEKTLKAWESVYQYWAVFMLDGMK

>Hamham_140_ALV_AHJH01002760.1 (Hammondia hammondi)

NLLFPLLAATGPKLTAHFYDRMFEHNPELKDVFNMNNQRNGDQ

RQALFDAICAYAANIENLAALLPAVERIAHKHTSLNIQPDQYQIVGGHLLATLDEMFSPG

QEVLDAWGKAYGVLANVFIQREDQDLSGQRSRRRWLA

>Linpol_156_ALV_JO746055.1 (Lingulodinium polyedrum)

GAREAAPPPPLTEDEAALVELTWGKAAALGAEEVGVLLFKHIFEIAPEALQLFSFKDEAD

LYSSPKLNRHGATVVKTVGAAVGGLGDLGGLAATLEALGKRHVGYAVRPEHYEVVGEALI

KTLRVGLADAFTPEAEAAWRKLYGLVSKTMIGTNYS

>Harver_143_AMOE_EC130120.1 (Hartmannella vermiformis)

RLRTSFTLVAKDNPVRITENFYDRLFRNHPEVRPMFPQDMSKQKMGLASALSLVVKSADN

LGKIEDVLLKMGARHVAYGTKDEHYGVVSSNIIATLKELSGDAWEAQWEQDWTNALNLVA

AVMIRGSNEFIAKQKAEQSTKSD

>Ectsil_161_STR_CBJ25775.1 (Ectocarpus siliculosus)

maepqaeghk aegevdvegy kaeirrtfal vepisvqaag ifyptlwevd tstkplfkdt

dmdkqgeklm ktlgvavaml nkmdtlkpil enlgrkhvdy gvtpemypsv gkallitfek

glgeectplt tkawtwvfgi issiciaaas evkpeekpee k

>Ectsil_180_STR_CBJ32473.1 (Ectocarpus siliculosus)

mfttraaree sykaevrrth allkpiqiea aaifyptlfe vdpstkplfk dvdmekqgak

lmkvigvavm mldkmdqfkp mlvklgkkhv tygvtddmyp svvsallitl ekglgeechq

ltkdawawvm nsiaavciaa aredtgapag

>Emihux_199a_ HAP_194900 (Emiliania huxleyi)

MPFPDTIPELRSSWSAAVGVLFFKRIFEIAPGALELFSFRDVPAPELYESAKLKSHALKV

MNTVGVAVSMLDDVPALLPVLEGLGKKHVDYGVVPAHYEVVGQALLDTLALGLGDAFTPE

LKAAWAEIYGVVSSTMIKGANKVPAPAYTE

>Emihux_230_HAP_420423 ((Emiliania huxleyi)

RTVQAVQASWAQAEALGLATVGALFFDALFATSASAEQL

FATQKFADGPAGRARFKLHTLNVMQTLSAAVYGLSDLGSLAPVLEALGESHLGYNVLHVH

YEAAGAALLATLRGGLGEEFTPALESAWREVYAFIAQSMLRGASRAMYTF

>Phycap_336_STR_PhycaF7|115153| (Phytophthora capsici)

HHQKEAKHITKAQNNKITELYDIFYAYLEEHGGDLKHVFRSSMHVRGRVL

VHISAGMRTMLASENIAEKILALTKTHRRFGVKLEHYDCVGRALLQAMEKVSGENWSPEI

DDAWRRMYSHSSVILIRTQKK

>Physoj_196_STR_EGZ28723.1 (Phytophthora sojae)

PPSSKSTNSGKRSQNSSGSTRITQLYDTFYSYLDHNSPALKPIFRSSMVVRSKVLVHISAG

LRTILTGDNMVERVESLTRTHLRFGVKMEHYDPLGVALIFAMKECSGALWSPQVEEAWRRL

YAHCCVILLDSHRRAMEQQATSKGREAGK

>Pytult_195_STR_PYU1T007201 (Pythium ultimum)

NIHMDARHTIGSGTPIGKLYQTFYEYLFEHYPHLKPLFQASIQIQSRVLVHISSGMKSLLSSEDL

VQKVMELALVHMKIGVAPEDFDPLGESLIQSMKITSGDDWNDQIERAWRRIYCHASILIL

VNIPNTTLDMGDFKD

>Pytult_234_STR_PYU1T007202 (Pythium ultimum)

DNSSSRKSSYESTAPSLIMIFYDTFYAYLERNSPDLKPVFRSSLHVRSKVL

VHISAGMRTLLATSSNFADRIESLTKTHLRFGVQMEFYNPLGLALFSAMKECSGDMWTPE

VENAWKRLFTHCSVLLLMHQKLTLERRFN

>Thaoce_209_STR_AGNL01013620.1 (Thalassiosira oceanica)

AYFEKRKEIIQSTWKTV

GDSLGVEATKLFYKRLFEEYPEVVPMFGDADMDKQAEKLLKTVSLAVEYLNDMGELVPIL

QGLGEKHAKEWKVKREHYAPVGASLLWTLETGLGEAWTEDTADAWTWVYGVIADTMADAG

>Thapse_154_STR_XP_002291928.1 (Thalassiosira pseudonana)

MGLSPEDLSLVQTSWAKVVPIASVAADLFYTKLFELDPELRPLFPSDLADQKKKLMAMIS

VAVDGLTDLEALVPAVQDLGRRHAKYYKVTSPMFDTVGAALLDTLEKGLGEGWDEEHKEA

WTLVYGVLSKTMIDAGEESTASDVDEK

>Galgal_GbE_151_NP_001008786.1 (Gallus gallus)

MSFSEAEVQSARGAWEKMYVDAEDNGTAVLVRMFTEHPDTKSYFTHFKGMDSAEEMKQSDQVRGHGKRVFTAINDMVQHLDNTEAFLGILNPLGQKHATQLKIDPKNFRIICDIILQLMEEKFGGDCKASFEKVTNEICTHLTNIYKEAGW

>Melgal_151_GbE_XP_003202379.1 (Meleagris gallopavo)

MSFSEAEVQSARGAWEKIYVDAEDNGTAVLIRMFTEHPDTKSYFTHFKGMDSAEEMKQSDQVRGHGKRVFTAINDMVQHLDNTEAFLGILNPLGQKHATQLKIDPKNFRIICDIILQLMEEKFGGDCKTSFEKVTNEICTHLTNIYKEAGW

>Xentro_200_GbX_NP_001011196.1 (Xenopus tropicalis)

MGCILSSLGWQWRDSLDHTETSPLLPTLNLSEQQQQLLVESWRLIQHDIAKVGVILFVRLFETHPECKDVFFLFRDVDDLQALRANKDLRAHGLRVLSFVEKSVARIADCARLEELALELGRSXYRYNAPPRYYQYVGTEFISAVCPMLHDKWTAEVEEAWKGLFAYICTVMERGYQEEERRHSDGRSLIDGLQGNKGLI

>Caraur_GbX_CAG25724.1 (Carassius auratus)

LTA NHIRLIKESW RLTQEDIAKV GIIMFVRLFE

THPECKDVFF LFRDVEDLER LRTSRELRAH GLRVMSFIEK SVARLDQLER LETLALELGK

SHYRYNAPPK YYGYVGAEFI CAVRPILKDR WTPELEEAWK TLFQYVTSIM REGF

>Tetnig_205_GbX_CAG25726.1 (Tetraodon nigroviridis)

MGCAISSLGAEAEFGDRSAEEEDAAAAAAVVYPREDQIQMIKDSWKVIRDDIAKVGIIMFVRLFETHPECKDVFFLFRDVEDLERLRSSRELRAHGLRVMSFIEKSVARLDQQDRLEALAVELGKSHYHYNAPPKYYSYVGAEFICAVQPILKERFTSELEEAWKTLFQYVTGLMRKGHQEEGSRQRHLALPPKDGPEKRTSAL

>Melgal_179_GbY_XP_003211469.1 (Meleagris gallopavo)

MEKVQGEMEIERWERSEEISDAEKKVIQETWSRVYANCEDVGVSILIRFFVNFPSAKQYFSQFKHMDDTLEMERSLQLRKHAQRVMGAINSVVENLNDPEKVSSVLALVGKAHALKHKVEPIYFKKLTGVMLEVIAEAYGNDFTPEAHGAWTKMRTLIYTHVTAAYKEAGWVSYPSATL

>Xenlae_156_GbY_NP_001089155.1 (Xenopus laevis)

MADLTAADIENINEIWCKIYANPEESGKTVVIRLFTTYPQTKVYFKNLKNIATLEEMQVNPGIRAHGKRVMGALNQVIQNLNDWEVVSSALTHLAQRHQDVHKVGVNNFQLLFLVILTIFKEALGADFTPEHCKSWEKLFSITYNFLDSCYTKSDS

>Ratnor_151_Ngb_Q8VH38 (Rattus norvegicus)

MERLESELIR QSWRAVSRSP LEHGTVLFSR LFALEPSLLP LFQYNGRQFS SPEDCLSSPE

FLDHIRKVML VIDAAVTNVE DLSSLEEYLA TLGRKHRAVG VRLSSFSTVG ESLLYMLEKC

LGPDFTPATR TAWSQLYGAV VQAMSRGWDG E

>Ornana_179_Ngb_XP_001508417.1 (Ornithorhynchus anatinus)

mengrlsgpe qelireswrs vnsnplehgm ilftrlfdle pdllplfqyn crqfssprdc

laspefldhi rkvmlvidaa vihlddlssl eeyltnlgrk hkaigvklss fstvgesllf

mlekclgpaf spatreawtr lytamvhams rgwggelnpa hihpqlhqhc shpqtpiln

>Tetnig_159_Ngb_tr|Q90W04 (Tetraodon nigroviridis)

MEKLSSKDKE LIRGSWDSLG KNKVPHGVIL FSRLFELDPE LLNLFHYTTN CGSTQDCLSS

PEFLEHVTKV MLVIDAAVSH LDDLHSLEDF LLNLGRKHQA VGVKPQSFAM VGESLLYMLQ

CSLGQAYTAS LRQAWLNMYS VVVASMSRGW AKNGEDKAD

>Macmul_190_Cygb_XP_001104888.1 (Macaca mulatta)

MEKVPGEMEI ERRERSEELS EAERKAVQAT WARLYANCED VGVAILVRFF VNFPSAKQYF

SQFKHMEEPL EMERSPQLRK HACRVMGALN TVVENLHDPD KVSSVLALVG KAHALKHKVE

PVYFKILSGV ILEVVAEEFA NDFPPETQRA WAKLRGLIYS HVTAAYKEVG WVQQVPNATT

PPATLPSSGP

>Anocar_193_Cygb_XP_003217236.1 (Anolis carolinensis)

MEKVQGEMEIERWERNEEMSDAEKKMIQETWNTVYAKCEDVGVSLLIRFFVNFPSAKHYFSQFKHMEDPLEMERSQQLRKHARRVMGAINSVVENIYDSEKVASVLALVGKAHAVKHKVEPVYFQILLGVLLEVLAEEYTNEFSPPEVQRAWAKMKSLICTHVTAAYKEEPPGDGGGSSSRSSSKVTEPFPQT

>Oncmyk_177_Cygb_CAD68070.1 (Oncorhynchus mykiss)

merqqgevta drlerldplc dseremikdt wakvyqncdd vgvailirlf vnfpsskqyf

sqfqqvedpg elersaqlrk hsrrvmnain tlvenlhdgd kmvsvlklvg kahalrhnve

pvyfkilcgv ilevlvadfp dyitpevava wtklldaiyw hvkgvyeevg wasssav

>Galgal_142_HbA_NP_001004376.1 (Gallus gallus)

MVLSAADKNNVKGIFTKIAGHAEEYGAETLERMFTTYPPTKTYFPHFDLSHGSAQIKGHGKKVVAALIEAANHIDDIAGTLSKLSDLHAHKLRVDPVNFKLLGQCFLVVVAIHHPAALTPEVHASLDKFLCAVGTVLTAKYR

>Caraur 143 HbA CAP69820.1 (Carassius auratus)

MSLSDKDKAVVKALWAKIGSRADEIGAEALGRMLTVYPQTKTYFSHWSDLSPGSGPVKKHGKTIMGAVGDAVSKIDDLVGALSSLSELHAFKLRIDPANFKILAHNVIVVIGMLFPGDFTPEVHMSVDKFFQNLALALSEKYR

>Ictpunc_143_HbA ADO29166.1 (Ictalurus punctatus)

MSLSAKDKAVVKDLWAKVAPKADDIGAEALGRLFEVYPQTKTYFSHWSDLTPGSAQVKKHGSVIVRKIGEAVGHIDDLTGALSSLSELHAFKLRVDPVNFKLLSHTIEVSIALFFPAEFTPEVHVSFDKFLQNLALALSEKYR

>Galgal_147_HbB_NP_990820.1 (Gallus gallus)

MVHWTAEEKQLITGLWGKVNVAECGAEALARLLIVYPWTQRFFASFGNLSSPTAILGNPMVRAHGKKVLTSFGDAVKNLDNIKNTFSQLSELHCDKLHVDPENFRLLGDILIIVLAAHFSKDFTPECQAAWQKLVRVVAHALARKYH

>Ictpunc_147_HbB_ADO29344.1 (Ictalurus punctatus)

MVVWTDFERATIQDVFSKIDYESVGHQALSRCLVVYPWTQRYFGSFGNLYNAAAIIGNPKVAAHGLVVVRGLEKAAKNMDNIKAIYADLSVLHSEKLHVDPDNFKLLADCITIVIASVLGASFTAEVQAALQKFLAVVVSALGKQYQ

>Caraur_148_HbB_CAP69821.1 (Carassius auratus)

MVEWTDAERSAIIGLWGKLNPDELGPQALARCLIVYPWTQRYFATFGNLSSPAAIMGNPKVAAHGRTVMGGLERAIKNMDNIKATYAPLSVMHSEKLHVDPDNFRLLADCITVCAAMKFGPSGFNADVQEAWQKFLSVVVSALCRQYH

>Musmus_154_Mb_NP_038621.2 (Mus musculus)

MGLSDGEWQLVLNVWGKVEADLAGHGQEVLIGLFKTHPETLDKFDKFKNLKSEEDMKGSEDLKK

HGCTVLTALGTILKKKGQHAAEIQPLAQSHATKHKIPVKYLEFISEIIIEVLKKRHSGDFGAD

AQGAMSKALELFRNDIAAKYKELGFQG

>Taegut_154_Mb_XP_002199416.1 (Taeniopygia guttata)

mglsdqewqq vltvwgkves dlaghghqil mrlfqdhpet ldrfekfkgl ktpdamkgse

dlkkhgvtvl tqlgkilkak gnheaelkpl aqthatkhki pvkylefise viikvlaekh

aadfgadaqa amkkalelfr ndmatkykef gfqg

>Carcar_153_Mb_P56208 (Caretta caretta)

GLSDDEWNHVLGIWAKVEPDLSAHGQEVIIRLFQLHPETQERFAKFKNLTTIDALKSSEEVKKHGTTVLTALGRILKQKNNHEQELKPLAESHATKHKIPVKYLEFICEIIVKVIAEKHPSDFGADSQAAMKKALELFRNDMASKYKEFGFQG

>Caraur_147_Mb_CAO00405.1 (Carassius auratus)

MADHELVLKCWGVVEADFEGTGGEVLTRLFKQHPETQKLFPKFVGIAQSDLAGNAAVNAHGATVLKKLGELLKARGDHAAILKPLATTHANKHKIALNNFRLITEVLVKVMAEKAGLDAAGQTALRKVMEAVIGDIDTYYKEFGFAG

>Petmar_149_AGN_P02208 (Petromyzon marinus)

PIVDTGSVAP LSAAEKTKIR SAWAPVYSTY ETSGVDILVK FFTSTPAAQE

FFPKFKGLTT ADQLKKSADV RWHAERIINA VNDAVASMDD TEKMSMKLRD

LSGKHAKSFQ VDPQYFKVLA AVIADTVAAG DAGFEKLMSM ICILLRSAY

>Eptbur_146_Q7SID0.1 (Eptatretus burgeri)

piidqgplpt ltdgdkkain kiwpkiykey eqyslnillr flkcfpqaqa sfpkfstkks

nleqdpevkh qavvifnkvn eiinsmdnqe eiikslkdls qkhktvfkvd siwfkelssi

fvstidggae feklfsiici llrsay

>Myxglu_Hb2_156_AGN_AAD40480.1 (Myxine glutinosa)

MSAHGIARTTEGERAAVRASWAVLMKDYEHAGVQILDKFFKANPAAKPFFTKMKDLHTLEDLASSADARWHVERIIQAVNFAVINIEDREKLSNKFVKLSQDHIEEFHVTDPQYFMILSQTILDEVEKRNGGLSGEGKSGWHKVMTIICKMLKSKY
