# Supplementary figures and images for: Hemoglobins in the genome of the cryptomonad Guillardia theta
Source: Biol Direct. 2014 May 8;9:7. doi: 10.1186/1745-6150-9-7 (PMC4101818; doi:10.1186/1745-6150-9-7)

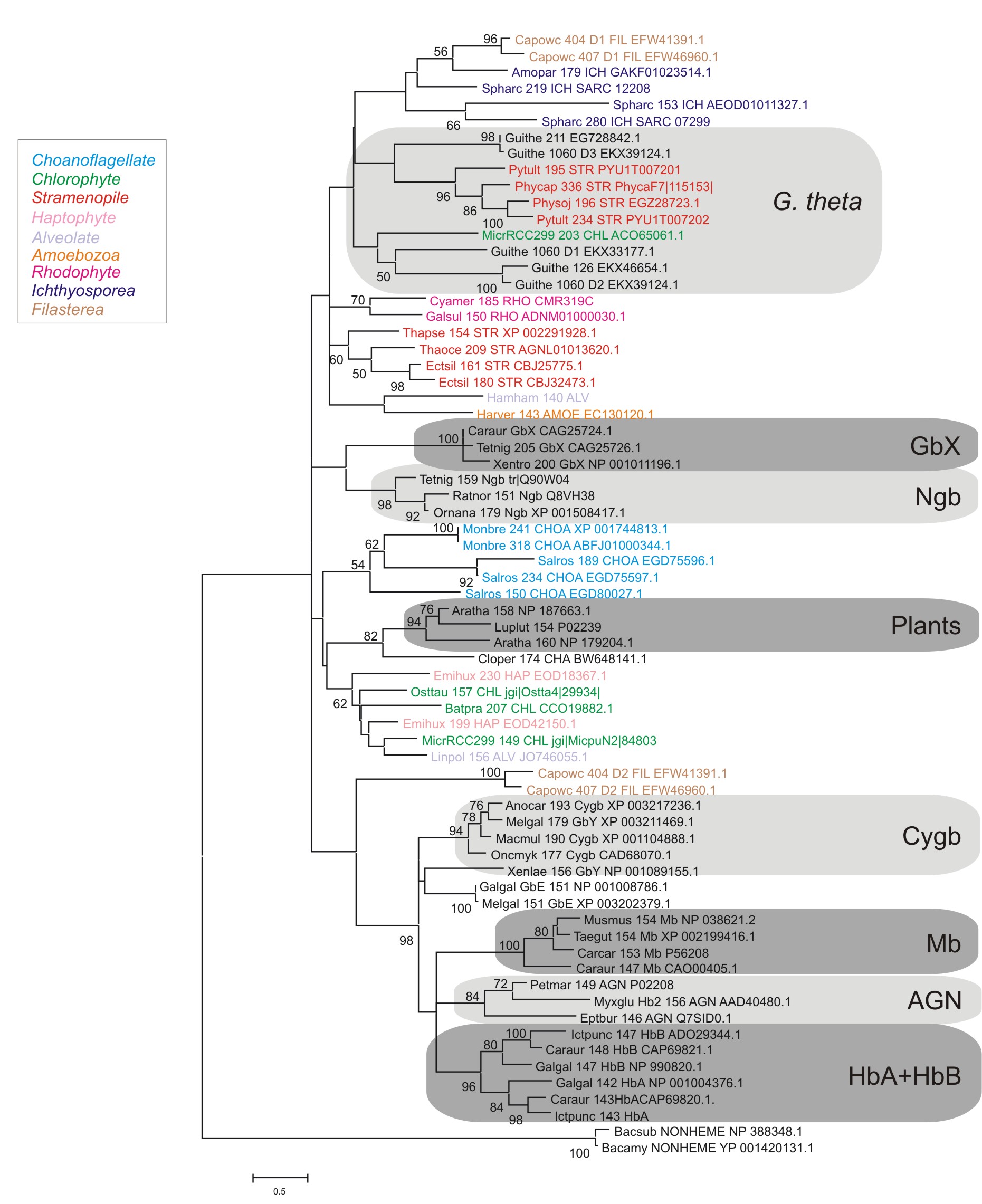

Supplement: Additional file 4 — Maximum likelihood tree of the Clustal Omega MSA of G. theta Hbs with representative vertebrate, protist, choanoflagellate Hbs and plant Hbs using two Bacillus nonheme globins as outgroup. ML analysis was performed by MEGA 5.2 under a WAG substitution model. The resulting trees was tested by bootstrapping with 100 replicates. Same sequences as in Figure 1B and Additional file 3. All globin sequences are identified by the first three letters of the genus name and the first three letters of the species name, the number of residues, the abbreviated phylum and family names, and their identification numbers. Support values at branches represent bootstrap percentages (>50) of ML analysis. Abbreviations of protist taxons: ALV – Alveolate; AMOE – Amoebozoa; CHOA – Choanoflagellates; CHL – Chlorophyte; FIL – Filasterea; ICH – Ichthyosporea; HAP – Haptophyte; RHO – Rhodophyte; STR – Stramenopile. [file 1745-6150-9-7-S4.jpeg]

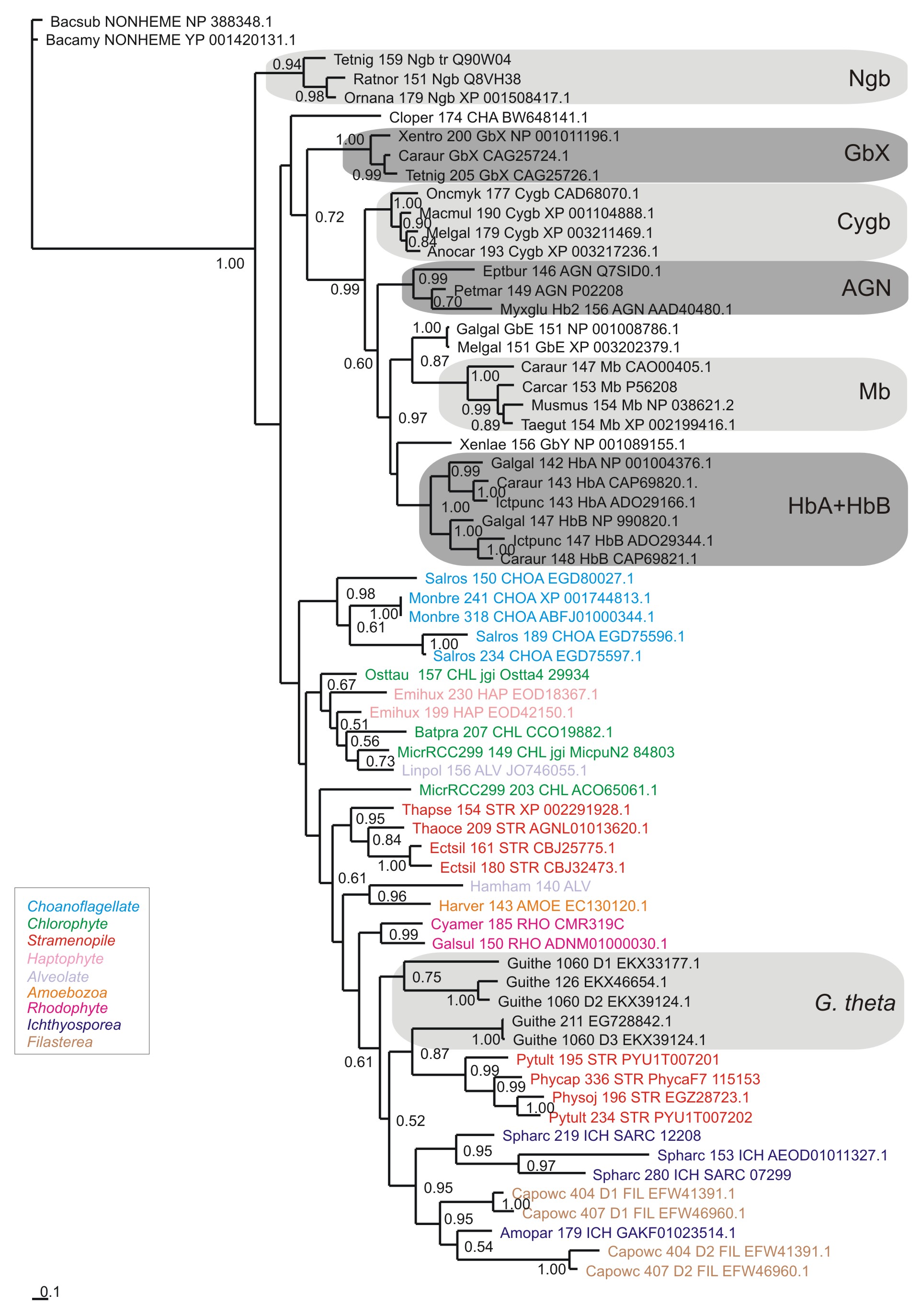

Supplement: Additional file 5 — Bayesian phylogenetic tree based on a MAFFT MSA, of G. theta Hbs with representative vertebrate, protist, choanoflagellate Hbs and plant Hbs using two Bacillus nonheme globins as outgroup. Bayesian phylogenetic reconstruction was performed by MrBayes 3.2.2 employing a mixed substitution model. MCMCMC sampling was carried out using 2 independent runs for 10′000′000 generations on the CIPRES web portal [39]. All globin sequences are identified by the first three letter of the genus name and the first three letters of the species name, the number of residues, the abbreviated phylum and family names, and their identification numbers. Support values at branches represent Bayesian posterior probabilities (>0.5). Abbreviations of protist taxons: ALV – Alveolate; AMOE – Amoebozoa; CHOA – Choanoflagellates; CHL – Chlorophyte; FIL – Filasterea; ICH – Ichthyosporea; HAP – Haptophyte; RHO – Rhodophyte; STR – Stramenopile. [file 1745-6150-9-7-S5.jpeg]

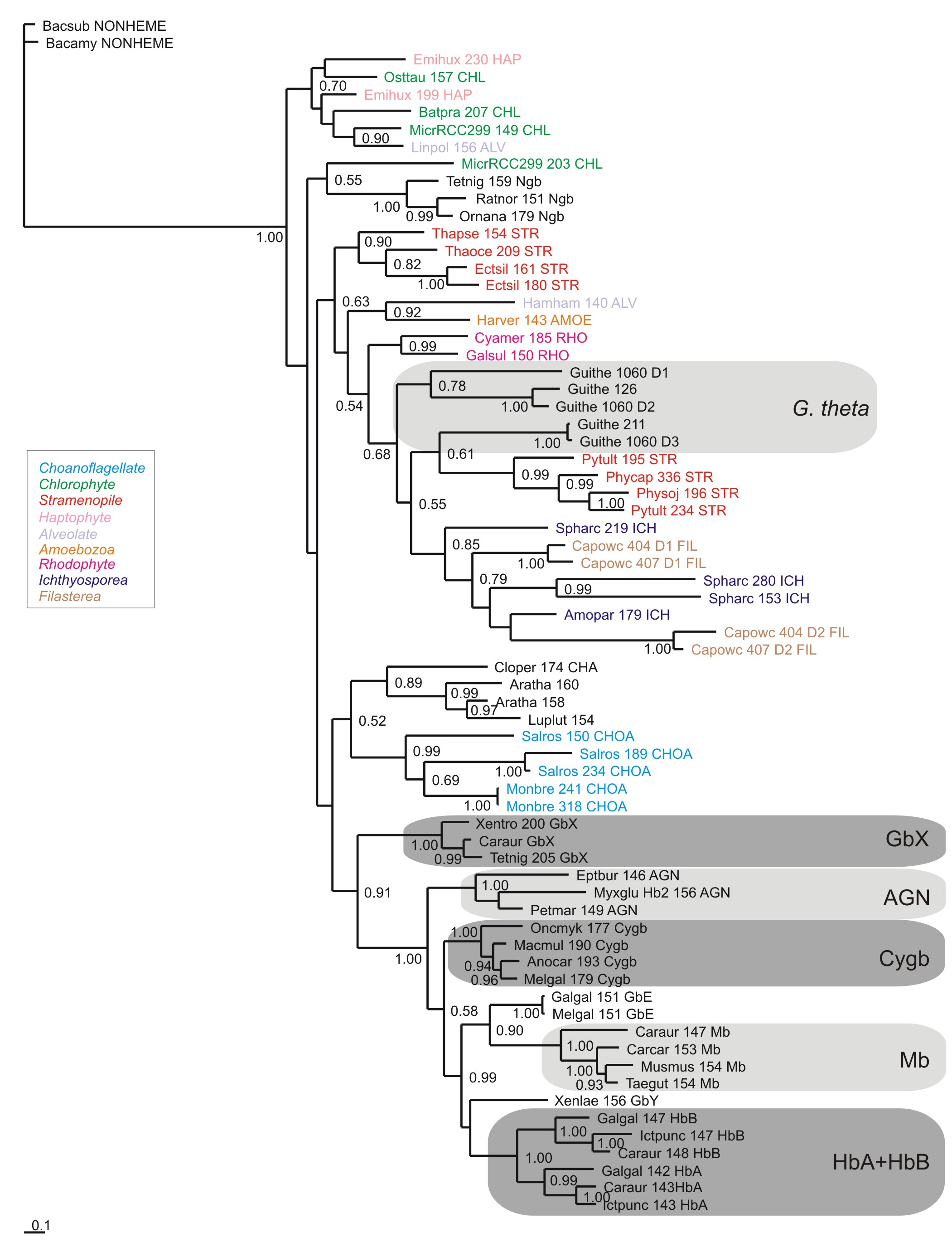

Supplement: Additional file 6 — Bayesian phylogenetic tree based on a MUSCLE MSA, of G. theta Hbs with representative vertebrate, protist, choanoflagellate Hbs and plant Hbs using two Bacillus nonheme globins as outgroup. Bayesian phylogenetic reconstruction was performed by MrBayes 3.2.2 employing a mixed substitution model. MCMCMC sampling was carried out using 2 independent runs for 10′000′000 generations on the CIPRES web portal [39]. All globin sequences are identified by the first three letter of the genus name and the first three letters of the species name, the number of residues and the abbreviated phylum and family names. Support values at branches represent Bayesian posterior probabilities (>0.5). Abbreviations of protist taxons: ALV – Alveolate; AMOE – Amoebozoa; CHOA – Choanoflagellates; CHL – Chlorophyte; FIL – Filasterea; ICH – Ichthyosporea; HAP – Haptophyte; RHO – Rhodophyte; STR – Stramenopile. [file 1745-6150-9-7-S6.jpeg]

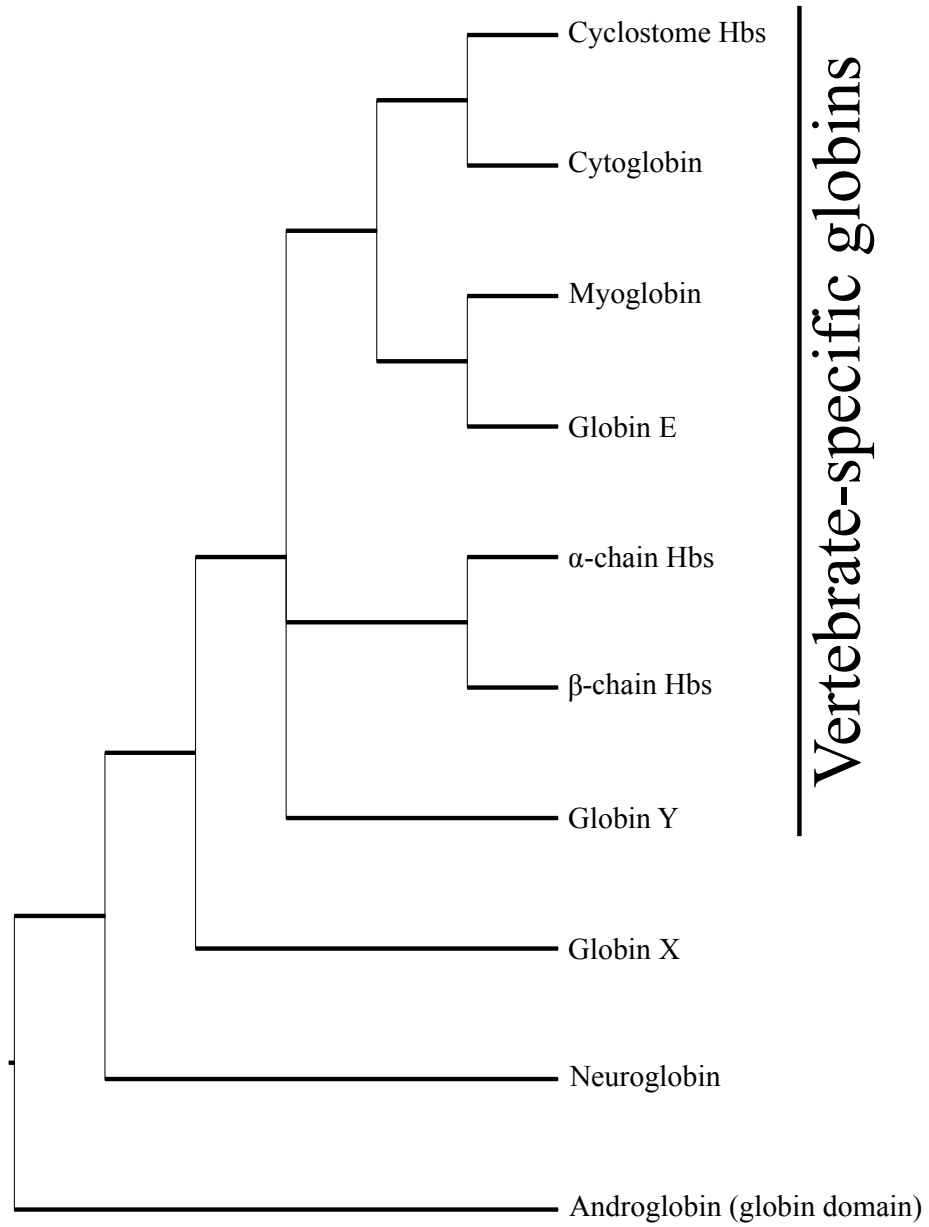

Vertebrate-specific globins

Supplement: Additional file 7 — A diagrammatic representation of the phylogeny of vertebrate globins. Figure one from ref [28]. [file 1745-6150-9-7-S7.pdf]
